# Patient Satisfaction with the Quality of Oral Rehabilitation Dental Services: A Comparison between the Public and Private Health System

**DOI:** 10.3390/dj12030045

**Published:** 2024-02-21

**Authors:** Cosmin Ionuț Lixandru, Ionela Maniu, Maria Mihaela Cernușcă-Mițariu, Mihai Iulian Făgețan, Ioan Sebastian Cernușcă-Mițariu, Horațiu Paul Domnariu, Magdalena Lixandru, Carmen Daniela Domnariu

**Affiliations:** 1 Faculty of Medicine, “Lucian Blaga” University, 550024 Sibiu, Romania; maria.mitariu@ulbsibiu.ro (M.M.C.-M.); mihai.fagetan@ulbsibiu.ro (M.I.F.); sebastian.mitariu@ulbsibiu.ro (I.S.C.-M.); magdalena.busu@ulbsibiu.ro (M.L.); carmen.domnariu@ulbsibiu.ro (C.D.D.); 2 Mathematics and Informatics Department, Faculty of Sciences, Research Center in Informatics and Information Technology, “Lucian Blaga” University, 550024 Sibiu, Romania; 3 Research Team, Pediatric Clinical Hospital Sibiu, 550166 Sibiu, Romania; 4 Doctoral School of Medicine, University of Oradea, 410087 Oradea, Romania; domnariu.horatiupaul@student.uoradea.ro

**Keywords:** oral rehabilitation, patient satisfaction, public health, private health, Dental Practice Questionnaire (DPQ)

## Abstract

Measuring satisfaction can help us understand patients’ expectations and adopt individualized treatment according to their expectations. In the current study, we applied the DPQ (Dental Practice Questionnaire) to analyze the degree of patient satisfaction regarding medical services in the public and private sector in a Romanian city from the central region. A group of 200 patients, 100 patients from the public sector and 100 patients from the private sector, participated in the survey. The results showed significant differences in response when patients were stratified by age, gender, visit frequency and length of time attending the same practice. Significant differences between public and private practices were encountered. Moreover, the degree of patient satisfaction was found to be related to appointment promptness/length of time and the confidentiality/ability to listen/knowledge/respect shown by the dentist, while patients’ recommendations to others were influenced by dentists’ explanations and warmth, followed by the appointment system and confidentiality. Patient satisfaction with oral rehabilitation dental services plays an essential role in maintaining patients’ addressability, but there is a multitude of factors that can influence patients’ opinions. Further analysis of the evolution of the influencing factors (causing satisfaction or dissatisfaction), in time, could provide deeper insights into the links between patient satisfaction and these factors.

## 1. Introduction

Patient satisfaction is a multifactorial concept that plays a major role in the addressability of patients to dental medical services. Busby et al. [1] structure the notion of success in dental practice in four areas: oral health, patient satisfaction, professional satisfaction of the medical staff and financial income. It emphasizes the idea that a higher degree of patient satisfaction can positively influence all other areas. Patient satisfaction should be one of the main goals for healthcare providers, as increased patient satisfaction can lead to higher clinic revenue, better clinic publicity, improved reputation and rank and (very) good trust in the doctor among the people. At the opposite pole, there is the possibility that general patient dissatisfaction leads to problems such as lower financial receipts, difficulties in maintaining the job market or difficulties in attracting new patients to the clinic [2]. Healthcare providers should focus on the patient. The patient must benefit from a proper medical consultation, a correct and well-argued diagnosis and a specific treatment, as well as very good communication with the medical staff. Also, the patient should be free to make their own choices, be treated with respect and fully benefit from the confidentiality of medical data. Any aspect that does not put the patient at the center of attention is correlated with a decrease in the quality of the medical service provided and, implicitly, with a decrease in patient satisfaction [3].

The quality of oral rehabilitation medical services is closely related to the level of patient satisfaction, a degree of satisfaction that must be evaluated by dentists in order to increase the quality of the medical services provided. The quality of the medical act is closely related to the relationship between the medical staff and patients, a relationship that needs to be adapted to the new times and in which the patient must always feel that they are the first priority [4]. A review on the topic of trust in the dentist–patient relationship concluded that there are still many things to improve, especially in the degree of trust of the patient towards the doctor and in the active role that the dentist should have in the doctor–patient relationship [5]. It has been reported that among the main factors that can influence the degree of satisfaction of patients with oral rehabilitation medical services are the professional training of the dentist, the clinical setting, accessibility and the overall appearance of the reception area. Patient satisfaction is a particularly important element in everyday medical practice and represents an aspect that can easily be improved [6]. It has often been stated that satisfaction is closely related to the concept that defines the “expectations” of patients, a dynamic and multidimensional concept by definition. This makes it difficult to measure the degree of satisfaction, as it is influenced by various factors, such as the individual characteristics of the patients, their belief system or their status before starting a medical treatment [7]. The study [8] stated that the degree of patient satisfaction is most frequently influenced by certain interpersonal factors, such as the patient’s perception of the attitude and behavior of the medical staff. Professional competence is not always the main benchmark for patients, and a lower degree of satisfaction among patients can be reflected in the referral to another dental practice, which demonstrates the special importance that this kind of study has in the continuous improvement of medical services provided by identifying the most important influencing factors of patient satisfaction. The permanent study of patients’ satisfaction with medical services is an essential element in the process of permanent improvement in the medical services offered to patients [9].

Measuring satisfaction can help us understand patients’ expectations and adopt individualized treatment according to their expectations. Over time, a multitude of quantitative questionnaires have been developed and applied to determine the degree of patient satisfaction with dental medical services [5,10], such as the 19-item Dental Satisfaction Questionnaire (DSQ) [11], the 10-item Dental Visit Satisfaction Scale (DVSS) [12], the 22-item Scale for Measuring Consumer Perception of Service Quality (SERVQUAL) [13], the 31-item Australian Dental Satisfaction Scale (DSS) [14], CAHPS Dental Plan Survey from AHRQ [15,16] and the Dental Practice Questionnaire (DPQ) [17]. The Dental Practice Questionnaire measures patients’ actual experiences (examines important aspects of the quality of dental care from the patient’s perspective rather than assessing the patient’s expectations or attitudes) and was designed specifically for the Practice Accreditation Scheme as part of the Australian National Safety and Quality Health Service [6,16,17].

However, there are not many studies (according to our knowledge) on the Romanian population regarding the measurement of patient satisfaction with dental medical services. Patient satisfaction with oral rehabilitation dental services plays an essential role in maintaining patients’ addressability, but there are a multitude of factors that can influence patients’ opinions. For this purpose, in the current study, we applied the DPQ to analyze the degree of patient satisfaction with medical services in the public and private systems in a Romanian city from the central region.

## 2. Materials and Methods

The present study is a cross-sectional study, implemented in March–April 2023, comprising a group of 200 patients, of which 100 patients were from the public system and 100 patients were from the private system. Patients who took part in the study are patients of the Emergency County Clinical Hospital in Sibiu and of a private clinic (Confort Dental) who have benefited from at least one dental consultation in the last year. The included patients who were over 18 years old, lived in Sibiu County, Romania, and gave their consent to participate in the study; we excluded patients under the age of 18 and those who did not agree to participate in the study. This study was approved by the Scientific Research Ethics Committee of the “Lucian Blaga” University of Sibiu through ethical approval number 33/2023. Patients’ consent regarding participation in the study was represented by the voluntary completion and return of a completed DPQ.

The study was carried out using the survey method based on a questionnaire. In the study, we used the DPQ (Dental Practice Questionnaire) [17]. The DPQ is a self-assessed questionnaire designed based on three dimensions: 7 performance evaluation questions are about access to the practice—‘dental practice’ performative items (Q1–Q7), while one question is about overall satisfaction with this visit to the dentist (Q8—‘summative’ patient satisfaction), 11 performance evaluation questions are about interpersonal and communication skills of the dentist—dentist ‘performative’ items (Q8–Q19), while one question is regarding dentist recommendations given by patients (Q20), and 3 questions are about the service provided at the practice (Q21–Q23). Items from the satisfaction scale (first 20 items) were measured on a Likert scale from 1 to 5 (1—low; 2—moderate; 3—good; 4—very good; 5—excellent), while items for questions about the service provided at the practice were binary (yes/no) [17].

The data were analyzed using descriptive analysis, correlations and regression analysis [18,19]. For each individual item of the questionnaire, the mean scores and 95% CIs (confidence intervals) were computed. Differences between groups (public vs. private system) were analyzed using parametric or nonparametric tests. Regression analysis was used to model the relationship between the dependent variables: ‘summative’ patient satisfaction (Q8), dentist recommendation given by patients (Q20) and the independent variables—(i) dental practice performative items (Q1–Q7) and (ii) dentist performative items (Q8–Q19).

## 3. Results

We analyzed 200 completed questionnaires from dental patients who received dental services in the public (100) and private (100) sectors. Socio-demographic characteristics of respondents are presented in Table 1. The descriptive statistic revealed that respondents were almost equal in terms of gender; in the whole group, 51.5% were females, but there were differences between the two sectors: in the public sector, 58% were males, whereas in the private sector, 61% were females. The mean age for the whole group was 42.19 years, ranging from 18 to 78 years old and mainly from the 25–44 (58%) age group, with no significant differences between the two subgroups. A total of 55.5% of the respondents were from urban areas (44% in the public sector vs. 67% in the private sector), and more than half (60%) graduated from higher education. The results showed that 87.5% of the patients went to a dentist they knew, and if we compare the responses from the public system versus the private system, we notice that the percentage is even higher (private 91% vs. public 84%). From the point of view of the period of time in which the patients attended the medical center, it should be noted that of the total number of respondents, 57.5% reported that they have been attending the medical center for a period between 5 and 10 years, with the lowest percentage being 15% for an interval of less than 5 years. Comparing the public system versus the private system, it can be seen that the answers are similar, but it can be noted that in the category of more than 10 years of attending the respective medical center, we find a higher percentage of patients who presented themselves in the private system (private 33% vs. public 22%).

The analysis of responses from the DPQ revealed that the highest scores on satisfaction items (first 20 items) were for Q19 (M = 4.21, SD = 0.88)—communication between the dental specialist and other staff, Q15 (M = 4.17, SD = 0.96)—respect shown by the dentist, Q13 (M = 4.16, SD = 0.94)—the dentist’s capacity to take into consideration the patient’s opinion regarding treatment options and Q9 (M = 4.16, SD = 1.02)—warmth of the dentist’s greeting of his patients. The lowest satisfaction levels were recorded for Q1 (M = 4.10, SD = 1.06)—satisfaction regarding the possibility of contacting the dental office by phone, Q6 (M = 4.09, SD = 1.07)—the ambiance and convenience of the waiting area and Q3 (M = 4.05, SD = 1.07)—fulfillment regarding the amount of time before routine appointments can be made.

On the satisfaction scale, lower average scores were reported for respondents who attended public clinics compared to those who went to private practices. The mean score and 95% CI on each item, for all responses and for the public and private sectors, are presented in Table 2.

Differences in mean scores of 0.20 or more were encountered in Q4 (*p* = 0.034), Q5 (*p* = 0.073), Q6 (comfort and adequacy of the waiting area) (*p* = 0.141), Q7 (*p* = 0.135) and Q16 (*p* = 0.092), while smaller differences were observed for Q17, Q13, Q18, Q15 and Q12.

**Figure 1:**
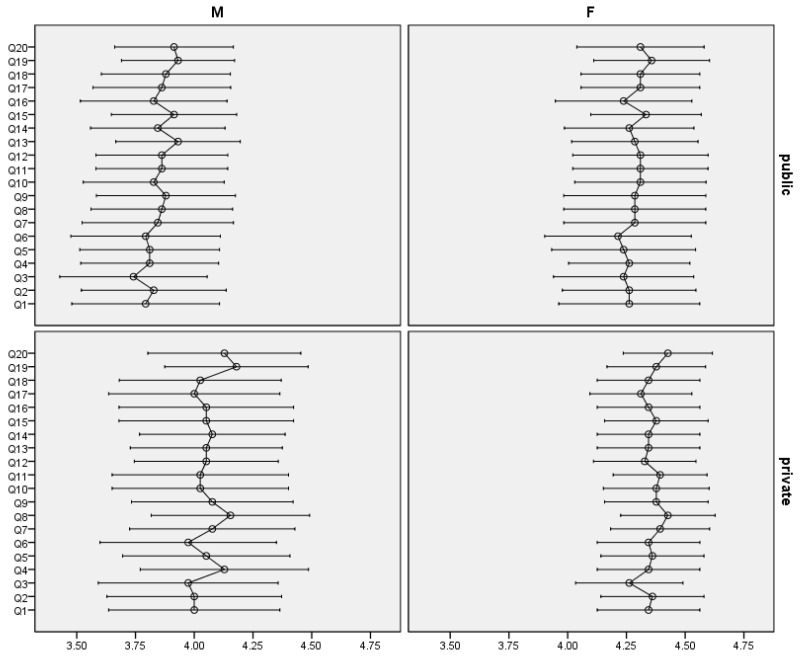
Mean score and 95% CI for individual items in public and private sector by gender.

Regarding gender (Figure 1), significant differences (*p* < 0.05) in responses were found for all items; male patients gave significantly lower scores than female patients (*p*-values ranging from 0.002 to 0.010). Going further with the analysis on each of the two sectors, it is observed that in the public sector, these significant differences are preserved in almost all items (marginal significant differences in the items waiting area (Q6, *p* = 0.068), how patients were treated by the reception staff (Q5, *p* = 0.062), patients’ involvement in choosing treatment options (Q13, *p* = 0.068) and confidence in the doctor’s abilities (Q16, *p* = 0.065). In contrast, in the case of the private sector, male patients gave lower scores than female patients on all items, but the differences were not statistically significant (*p* > 0.05).

**Figure 2:**
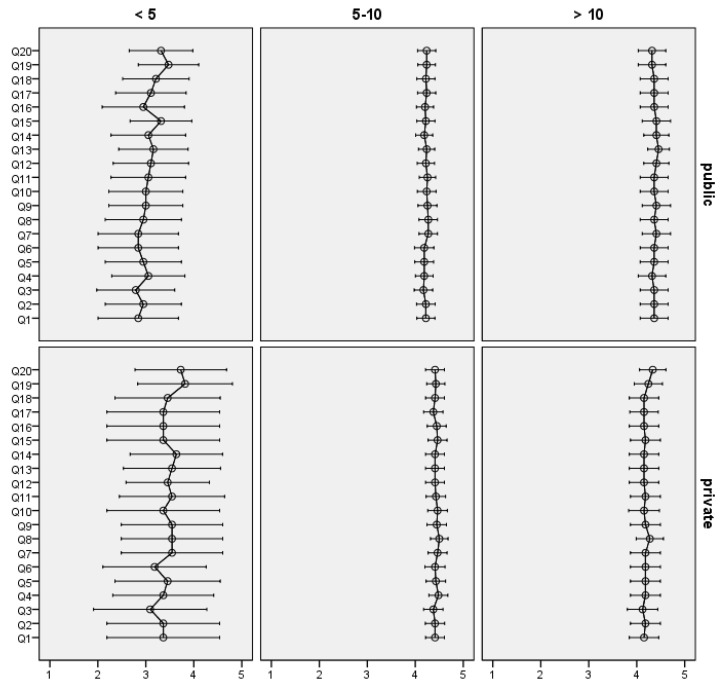
Mean score and 95% CI for individual items in public and private sector by time attending the practice.

Patients that have visited the same practice for more than 5 years reported significantly higher scores on all items when compared with patients who visited the same practice for less than 5 years (Figure 2). For the first two categories of the item regarding the number of years attending the same practice (<5 years, 5–10 years), patients who attend a public clinic gave lower scores than patients who attend a private practice, but the differences were not statistically significant except for in the 5–10 years category; these items included Q4—satisfaction regarding emergency appointments (*p* = 0.011), Q15—respect shown by the dentist (*p* = 0.038) and Q16—confidence in this dentist’s ability (*p* = 0.032). For the category over 10 years, an inverse situation can be observed, with patients from the public sector offering higher scores than those from the private sector for all items (but the differences were not statistically significant, *p* > 0.05).

Patients visiting their usual dentist gave significantly higher scores on all items (with mean differences ranging between 1.24 and 1.71), regardless of sector. As the general trend, respondents from the private sector, both those who visit their usual doctor and those who do not, gave significantly higher scores for all items when compared with respondents from the public sector. 

Six stepwise regression models (M1–M6) were constructed to identify the most important items influencing ‘summative’ patient satisfaction (models M1–M3; in these models, the dependent variable was Q8—overall satisfaction with this visit to the dentist) and ‘summative’ dentist recommendations (models M4–M6; in these models, the dependent variable was Q20—the recommendation you would give your friends about this dentist). Models M1 and M4 were performed using the entire dataset (public and private sectors), models M2 and M5 were performed using data from the public sector and models M3 and M6 were performed using data from the private sector. The results of applying the regression models are presented in Table 3. In the case of the entire dataset (regardless of the sector), the most important items affecting patient satisfaction were Q7, Q18, Q15, Q17 and Q4, and the most important items affecting dentist recommendation were Q11, Q2, Q18 and Q9. The influencing items for public and private sectors were different (Table 3), with the exception of the following items: Q7 (satisfaction with the promptness of being seen when you attend for an appointment), which is an influence item for overall visit satisfaction both in the public and private sector, and Q11 (dentist’s explanations of things to you), which is an influence item for dentist recommendation both in the public and private sector.

## 4. Discussion

The degree of patient satisfaction can help to identify the positive and negative aspects of medical centers and therefore can help to improve the quality of treatment and medical services provided to patients, with their feedback being an essential source of information regarding this aspect [12,20]. The main objectives of this study were to obtain a better understanding of how patients perceive the experience at the dentist and expose the differences regarding the degree of satisfaction of patients with medical services in the public versus the private system.

Our study results showed high patient satisfaction, reflected by average scores of all items greater than 4 (ranging from 4.05 ± 1.07 on item Q3 to 4.21 ± 0.88 on item Q19). Consistent with our results, a study [17] carried out using the DPQ on 58 dental clinics in Australia reported a mean of 4.66 for the aggregated scores of the analyzed practices. It can be observed that the mean row scores from our study are smaller than in Narayanan and Greco’s study (ranging from 4.30 on the Q3 item to 4.79 on items Q5 and Q16). In a study [21] on dental clinics in Saudi Arabia, using a personalized questionnaire [22] (structured in four areas: doctor–patient interaction, professional competence, the efficiency of the administrative system and the organization of the clinic), it was found that 79.5% of patients were overall satisfied with the quality of the medical services, with most of them (98.1%) also being satisfied with the doctor’s concentration during the medical procedure and his friendly attitude. Also, a study from India [23] (using a questionnaire consisting of 22 questions) showed that most patients (89%) were overall satisfied with the quality of the medical services provided, but it should be highlighted that in the category of patients who reported a lower degree of satisfaction, their main dissatisfaction was related to the long waiting time required for consultation and specialized treatment.

The results of our study are similar to the results of the study conducted by Armfield et al. [24]; the highest scores regarding the reasons behind the high degree of satisfaction were in relation to the items regarding the level of friendliness shown by the dentist (69.5%), the dentist’s availability to provide explanations (60.3%), the level of friendliness shown by the rest of the medical staff (51.1%) and the respect given by the dentist to his patients (40.2%). A study from Switzerland [24] also pointed out that in Western countries, patients carry out a lot of research to find the right dentist, so this may be the main reason why, in these countries, patients report high levels of satisfaction with services of oral rehabilitation. One of the main concerns from the point of view of addressability to oral rehabilitation services is the so-called “fear of the dentist”. These patients will generally report lower levels of satisfaction with dental services compared to patients who have a lower degree of fear of the dentist. This causes these patients to delay or even avoid visits to the dentist and thus present poorer oral health. A cross-sectional study [6] conducted in Iraq showed that at least half of the patients were satisfied with the quality of the medical services provided. The best results were in the reception area (70.4%), in the clinical setting (65.5%) and among the reception staff (62.7%). Overall, the percentage of patients satisfied with the quality of medical services was 57.7%, a much lower percentage compared to our study. Study [25] reported the best degrees of satisfaction among patients in aspects related to the time of waiting, the behavior of the doctor and the staff within the medical center, as well as the measures to prevent possible infections. On the other hand, the most unsatisfactory scores were reported for aspects related to the cost of the medical service, the social assistance service and the waiting time in order to start specialized treatment. Study [26] concluded that the degree of satisfaction is lower among younger patients and patients who have visited the dental service during the last year, as well as among patients whose reason for a visit to the dentist stemmed from pain.

Significant differences between public and private practices were encountered in our study. Respondents who had attended public clinics recorded significantly lower satisfaction scores than those who had attended private practices. In both sectors, high levels were encountered on items regarding the way in which the dentist communicates with other staff (Q19), and low levels were found on items Q3—satisfaction with the length of time before routine appointments can be made and Q6—comfort and adequacy of the waiting area.

Patients from the public system were treated in a University Hospital, where the treatments were being performed by students, resident doctors, specialist doctors and primary care doctors. In comparison, patients in the private system were mainly treated by specialist doctors and primary care doctors. Taking into account this aspect, some dissatisfactions of patients in the public system, such as the way of communication or the long duration of therapeutic procedures, may be due to the fact that a good number of them were treated by students and resident doctors who have less experience compared to a specialist or primary care physician.

On the other hand, the University Hospital, which has the status of an Emergency Hospital, can contribute to increasing the degree of patient satisfaction in some aspects, such as the quality of emergency treatment, the reduced waiting time for emergency appointments, the promptness of the takeover by the medical staff, the accessibility of the medical center or the low costs of specialized treatment. It should also be emphasized that the dental services provided by dental clinics in the public health system usually include essential treatments, such as prophylaxis treatments, dental fillings, endodontic treatments, extractions or other minor surgical interventions. On the other hand, dental clinics in the private medical system, depending on their investment in new equipment and technologies, as well as in the continuous training of medical personnel, can offer a much wider range of medical services, including complex orthodontic treatments, oral implants and even dental and facial aesthetics.

A review study [27] aiming to make a comparison between public and private healthcare systems in low- and middle-income countries came to the conclusion that the public sector often seems to lack promptness and hospitality towards patients. This aspect is also noticed in our study, with the lowest scores in the public system being encountered in the items related to contacting the office, the long period of obtaining an appointment, the environment of the waiting room and the hospitality of the reception staff. Moreover, the best scores obtained in our study in the private system are precisely related to the items that define the way patients are greeted, the promptness of the consultation and the way the doctor interacts with the patients and medical staff. The study [28] mentioned that oral rehabilitation medical services in the public system are generally preferred by patients due to the medical insurance they can benefit from, although their expectations regarding the quality of medical services provided in the public system are not the highest. Amorim et al. [29] mention that oral health services provided in the public system play an important role in reducing social inequities through access to medical services and socially disadvantaged groups. Macarevich et al. [30] highlight the fact that one of the advantages of dental medical systems in the private sector is that, in general, they can provide various therapeutic procedures, such as orthodontics or aesthetics, which cannot be provided in the public system. Also, the superior infrastructure in the private system may be a factor that determines a higher level of satisfaction among patients, especially among adolescents. Instead, through national programs to improve dental services in the public system, which may include the expansion of practiced procedures or the settlement of as many services as possible, the differences regarding the degree of satisfaction between the public system and the private system tend to decrease.

A study [8] conducted in Saudi Arabia revealed that patients who applied to the private medical system presented a 27% higher degree of satisfaction than patients who applied to the public medical system. The authors base this on the fact that dentists and other staff in private dental clinics are better professionally trained and have more experience, and the strict appointment system used in the private medical system helps the doctor to take care of the scheduled patient much better. The most favorable things that determined the highest degrees of satisfaction were related to hygiene and order in the office, privacy during treatment and the behavior of the dentist.

The research in [17] (also using the DPQ) reported that patients under the age of 25 had a lower degree of satisfaction compared to patients in other age groups. The study [9] revealed that patients over 39 years of age reported a lower degree of satisfaction than younger patients. This may be due to the fact that, in general, young patients have better oral health than older patients, and all treatments performed on patients who already have a high level of oral health are much less invasive; thus, patient feedback is more satisfactory. The study [24] reported that the degree of patient satisfaction was higher among older age groups (but this was not statistically significant). The results of the studies [8,29,30,31,32] also showed a higher degree of satisfaction among older patients. The lower levels of satisfaction found among young people can be attributed to their higher demands and very high expectations from the point of view of the medical act (expectations that cannot always be met to the standards they expect) [32]. However, patient age also plays an important role in studies [6,33], where overall satisfaction with dental services decreases with age. Also, satisfaction with the performance of the dentist and with the accessibility and reception staff decreases with older age.

In the case of gender, the results of our study showed that males gave lower scores than females on all items regardless of sector. In the public sector, males gave significantly lower scores on programming mode (Q1, Q2, Q3 and Q4), communication with the doctor (Q10, Q11, Q12 and Q17), ethical and professional conduct of the doctor (Q14, Q15, Q18 and Q19) and the tendency to recommend that doctor to friends (Q20). However, in the public sector, the differences remained but were not significantly significant. From this point of view, the results are consistent with those obtained from studies [9,17,24]. A possible explanation of the fact that female persons show a higher degree of satisfaction with oral rehabilitation services than male persons could be due to the fact that women visit dental services more often, which lowers their expectations. Lower satisfaction among men (in comparison with women) regarding accessibility, reception area and reception staff was also reported in Hussein’s study [6], with results that were similar to our findings. In contrast, there are studies [33,34] reporting a higher degree of satisfaction among men, an aspect attributed to possible higher expectations of medical services among women.

The tendency for patients with a higher level of education to report a higher degree of satisfaction with oral rehabilitation medical services is reported in the study [24], in which patients with university education and patients with higher vocational school report significantly higher scores compared to patients with primary/secondary education, high school/gymnasium education or with professional school education. This tendency is also reported in the studies [23,35]. In contrast, other studies [6,9,33,36,37] revealed that patients with higher education reported lower levels of satisfaction with dental services, possibly due to a higher expectation level. In the study of Amorim [29], patients with a lower level of education show a higher degree of satisfaction, an aspect that they do not consider relevant due to the fact that a lower level of education can create a false opinion of patients regarding the quality of the medical services they have benefited from. The study [8] also revealed a significant difference in the level of satisfaction of patients in relation to their level of education, with those with higher education reporting lower scores, an aspect attributed to the fact that they can analyze the experience they have in a medical clinic more objectively. In addition, among the conclusions of this study was the fact that the duration of the specialized treatment is recommended to be as short as possible so as not to cause the nervousness of the patients at a given moment, and a smile must be constantly used in the relationship with the patients. However, it is believed that patient satisfaction can be achieved through effective communication by both parties involved.

In our research, patients who attended the practice for more than 5 years gave significantly higher scores on most items. These findings are consistent with those reported in other sociodemographic studies [14]. In the study [17] (also using the DPQ), one of the conclusions was that patients who attended the dentist regularly or had attended for more than 10 years reported more favorable scores for most of the questions formulated in the DPQ. The study carried out by Tamaki [38] in Japan revealed that the attendance rate of a certain medical center for oral rehabilitation, as well as the periodic checks at the dentist, have an important influence on the degree of patient satisfaction, with almost half of the patients included in the study reporting a very high degree of satisfaction and confidence in the periodic checks they perform in their favorite medical centers.

The regression models from this study show that the degree of patient satisfaction is influenced by the appointment promptness/length of time and the confidentiality/ability to listen/knowledge/respect shown by the dentist. At the same time, a patient’s recommendations to others are influenced by the dentist’s explanations and warmth, followed by the appointment system and confidentiality. The study [17] demonstrated that the degree of patient satisfaction is influenced, in particular, by the interaction between the patient and the medical staff and by the level of communication between them, as well as by accessibility. The study [39] revealed that the main causes that can be the basis of a low degree of satisfaction among patients can be the long waiting time for treatment and the low level of knowledge of the medical staff regarding the reasons for the patient’s presentation to the doctor, as well as the lack of detailed explanations given by the dentist regarding the treatment options. Another factor that can influence the degree of patient satisfaction, according to the study [29], is a well-established work schedule at the level of the dental office, which is adapted to the daily schedule of the patients. It was also highlighted that there are a number of studies that show that a greater degree of satisfaction among patients can be obtained by explaining to them specific methods of prophylaxis of possible problems in the oral cavity, as well as by documenting the patient’s dental status on the basis of their dental records and monitoring it at each visit.

The results of this study highlight the fact that there is a need to make some improvements to oral rehabilitation dental services, especially in the public system. The decision makers in the field of health should take into consideration new measures to improve the services offered from the point of view of the accessibility of dental services and reducing waiting times for appointments and interventions. At the same time, innovative solutions are needed in terms of the costs associated with dental services, such as the implementation of dental health insurance schemes for vulnerable categories. Also, the findings of our study show that the doctor–patient relationship and open communication are important pillars for improving the quality of dental services. Summarizing, our study highlights the fact that accessibility, competence of medical staff, cost management and effective communication are essential factors that must be addressed to transform the public oral health system into a solid pillar of the general well-being of the population.

This study has some limitations. The sample size of our study was small, and it was conducted on patients from only one public and one private oral rehabilitation medical provider, so its generalizability is limited. Our sample was not homogeneous in relation to the sex of the participants of the study, which should be acknowledged when interpreting the results. The DPQ was applied only once on patients who benefited from at least one dental consultation in the last year; it was not applied again at a later time interval (when the medical treatment was completed). Thus, there is the possibility that the increased results in terms of the degree of satisfaction can also be attributed to the fact that complex procedures with a high degree of difficulty are not always required, and comfort during the visit to a dental center may be more important. The present study, being a cross-sectional study, only exposes the opinions of the patients at that moment, so the evolution of the influencing factors of satisfaction is not fully detailed, an aspect that will be improved in the future by in-depth studies carried out on larger groups of patients and over a longer time period. Also, a more in-depth study in the field should consider different categories of factors that could influence patient satisfaction with regard to the quality of oral rehabilitation dental services, assessing several public and private oral rehabilitation medical service providers. In the case of both systems, it is recommended to study patient satisfaction both before the start of treatment and at its completion in order to evaluate the evolution of the influencing factors of satisfaction, the long-term results of the treatment and the post-treatment services offered (including emergency care and consultation). Moreover, future studies should consider investigating the level of knowledge of patients regarding treatment options and their rights.

## 5. Conclusions

The study aimed to analyze the degree of patient satisfaction with medical services in the public and private systems among Romanian patients from a city in the central region. Satisfaction has been shown to have a complex relationship with multiple factors, such as age, gender, education level and other sociodemographic factors, and the current study provides a number of insights into the links between patient satisfaction and these factors.

The present study showed that oral rehabilitation medical systems, both from the public and from the private sector, report a high degree of satisfaction among patients, with higher values for the private system. At the level of both sectors, the best levels of satisfaction among patients were reported regarding items related to the communication between the dentist and both patients and medical staff. At the same time, the present study highlighted the fact that some dissatisfactions of patients in the public system are related to the method of communication or the long duration of therapeutic procedures, as well as the environment of the waiting room and the hospitality of the reception staff, indicating the need for both public and private oral rehabilitation medical centers to develop strategies to attract patients and provide complex treatment with a high degree of patient satisfaction.

## Figures and Tables

**Table 1:** Sociodemographic characteristics of respondents.

Characteristics	All 200	Public 100	Private 100	*p*-Value
Gender, n(%)				
male	97 (48.5)	58	39	0.007
female	103 (51.5)	42	61	
Age (years, mean ± SD) (range)	42.19 ± 10.15 (18–78)	42.23 ± 10.63 (19–78)	42.15 ± 9.71 (18–67)	0.956
Age groups				
<25	13 (6.5)	7	6	0.483
25–44	116 (58.0)	59	57	
45–65	66 (33.0)	30	36	
>65	5 (2.5)	4	1	
Residence				
urban	111 (55.5)	44	67	0.001
rural	89 (44.5)	56	33	
Education				
secondary education	80 (40.0)	44	36	0.248
higher education	120 (60.0)	56	64	
Consult the attending dentist				
yes	175 (87.5)	84	91	0.134
no	25 (12.5)	16	9	
How long have you been attending this dental center?				
<5 y	30 (15.0)	19	11	0.110
5–10 y	115 (57.5)	59	56	
>10 y	55 (27.5)	22	33	

**Table 2:** Mean scores of responses to individual items of the DPQ.

	DPQ	All	Public	Private	Mean Diff.	*p*
**Q1**	Your satisfaction with contacting the practice by phone	4.10 ± 1.06	3.99 ± 1.12	4.21 ± 0.98	0.22	0.151
**Q2**	Your satisfaction with the system for making appointments	4.12 ± 1.04	4.01 ± 1.09	4.22 ± 0.99	0.21	0.128
**Q3**	Your satisfaction with the length of time before routine appointments can be made	4.05 ± 1.07	3.95 ± 1.12	4.15 ± 1.02	0.20	0.189
**Q4**	Your satisfaction with the length of time before emergency appointments can be made	4.13 ± 1.00	4.00 ± 1.02	4.26 ± 0.96	0.26	0.034
**Q5**	The manner in which you were treated by the reception staff when attending the practice	4.12 ± 1.03	3.99 ± 1.09	4.24 ± 0.97	0.25	0.073
**Q6**	The comfort and adequacy of the waiting area	4.09 ± 1.07	3.97 ± 1.14	4.20 ± 0.99	0.23	0.141
**Q7**	Your satisfaction with the promptness of being seen when you attend for an appointment	4.15 ± 1.05	4.03 ± 1.14	4.27 ± 0.94	0.24	0.135
**Q8**	Your overall satisfaction with this visit to the dentist	4.18 ± 1.01	4.04 ± 1.09	4.32 ± 0.90	0.28	0.056
**Q9**	The warmth of the dentist’s greeting to you	4.16 ± 1.02	4.05 ± 1.08	4.26 ± 0.95	0.21	0.142
**Q10**	The dentist’s ability to really listen to you	4.14 ± 1.04	4.03 ± 1.07	4.24 ± 1.01	0.21	0.096
**Q11**	The dentist’s explanations of things to you	4.15 ± 1.00	4.05 ± 1.03	4.25 ± 0.96	0.20	0.117
**Q12**	The opportunity the dentist gave you to express your concerns or fears	4.14 ± 0.97	4.05 ± 1.03	4.22 ± 0.89	0.17	0.265
**Q13**	The dentist’s ability to involve you in choices about treatment options	4.16 ± 0.94	4.08 ± 0.96	4.23 ± 0.92	0.15	0.202
**Q14**	The sensitivity of the dentist when he/she examines you	4.13 ± 0.97	4.02 ± 1.02	4.24 ± 0.90	0.22	0.111
**Q15**	The respect shown to you by this dentist	4.17 ± 0.96	4.09 ± 0.93	4.25 ± 0.99	0.16	0.101
**Q16**	Your confidence in this dentist’s ability	4.12 ± 1.05	4.00 ± 1.10	4.23 ± 0.98	0.23	0.092
**Q17**	The information given to you to help you keep your teeth/mouth healthy	4.12 ± 1.00	4.05 ± 1.02	4.19 ± 0.97	0.14	0.284
**Q18**	Your confidence in the dentist’s respect for confidentiality	4.14 ± 0.96	4.06 ± 0.97	4.22 ± 0.95	0.16	0.174
**Q19**	The way in which this dentist communicates with other staff	4.21 ± 0.88	4.11 ± 0.89	4.30 ± 0.87	0.19	0.069
**Q20**	The recommendation you would give to your friends about this dentist	4.20 ± 0.91	4.08 ± 0.94	4.31 ± 0.86	0.23	0.057
	All items	4.14 ± 0.97	4.03 ± 1.02	4.24 ± 0.92	0.20	0.131

**Table 3:** Regression models.

	Q8			Q20		
	M1 (all)	M2 (Public)	M3 (Private)	M4 (All)	M5 (Public)	M6 (Private)
**Q1**	NS	NS	NS	NS	0.829 (0.006)	NS
**Q2**	NS	NS	NS	0.364 (0.006)	0.767 (0.000)	NS
**Q3**	NS	NS	NS	NS	NS	NS
**Q4**	0.126 (0.028)	0.111 (0.050)	NS	NS	NS	NS
**Q5**	NS	0.217 (0.018)	NS	NS	0.435 (0.050)	NS
**Q6**	NS	0.157 (0.042)	NS	NS	0.372 (0.047)	NS
**Q7**	0.845 (0.000)	0.852 (0.000)	0.959 (0.000)	NS	NS	NS
**Q9**	NS	0.260 (0.000)	NS	0.221 (0.045)	NS	0.488 (0.031)
**Q10**	0.212 (0.004)	0.390 (0.000)	NS	NS	NS	NS
**Q11**	NS	NS	NS	0.430 (0.000)	0.344 (0.036)	NS
**Q12**	NS	NS	NS	NS	0.264 (0.050)	0.364 (0.048)
**Q13**	NS	NS	NS	NS	NS	NS
**Q14**	NS	NS	NS	NS	NS	NS
**Q15**	0.136 (0.018)	0.341 (0.000)	NS	NS	NS	NS
**Q16**	NS	NS	NS	NS	NS	NS
**Q17**	0.136 (0.018)	NS	0.214 (0.008)	NS	NS	0.700 (0.010)
**Q18**	0.326 (0.003)	0.564 (0.000)	NS	0.379 (0.035)	0.450 (0.014)	NS
**Q19**	NS	NS	NS	NS	0.829 (0.006)	NS
**R^2^**	0.971	0.993	0.956	0.901	0.943	0.892
***p*-val.**	0.000	0.000	0.000	0.000	0.000	0.000

For models M1–M3, the dependent variable was Q8—overall satisfaction with this visit to the dentist; for models M4–M6, the dependent variable was Q20—the recommendation you would give your friends about this dentist; values represent the regression coefficients (*p*-value); NS—not significant (*p* > 0.05).

## Data Availability

The data are available on request from the corresponding author.
